# Electronic cigarettes and cardiovascular outcomes: a systematic review and meta-analysis of Major Adverse Cardiovascular Events (MACE)

**DOI:** 10.1186/s12889-026-26302-x

**Published:** 2026-02-23

**Authors:** Suraa N. Al-Rubaye, Mohammedsadeq A. Shweliya, Mohamed Fawzi Hemida, Mohammad Bdair, Amr M. Abou Elezz, Mohamed Ashraf Shehab, Yara Abukhaled, Abdulrahman Raad Abdulkareem Al-waeli, Al-Tuaama Abdullah Zeyad Hameed, Abbas F. Abdul Hussein, Khadeeja Ali Hamzah, Mustafa Al-Jarshawi

**Affiliations:** 1 Imam Al-Sadiq Teaching Hospital, Coronary Care Unit, Hillah, Babylon Iraq; 2 University of Baghdad College of Medicine, Baghdad, Iraq; 3 Faculty of Medicine, Alexandria University, Alexandria, Egypt; 4 Department of Medicine, Faculty of Medicine and Health Sciences, An-Najah National University, Nablus, Palestine; 5 Faculty of Medicine, Tanta University, Tanta, Egypt; 6 Faculty of Medicine, Menoufia University, Menoufia, Egypt; 7 The University of Tennessee Health and Science Center, Memphis, TN USA; 8 Department of Internal Medicine, Al-Karkh General Hospital, Baghdad, Iraq; 9 Privolzhsky Research Medical University, Nizhny Novgorod, Russia; 10 Babylon University, College of Medicine, Hilla, Babylon Iraq; 11 Baghdad university Alkindy College of Medicine, Baghdad, Iraq; 12 Faculty of Medicine & Health Sciences, Keele Cardiovascular Research Group, Keele University, Newcastle-under-Lyme, UK; 13 Institute of Health Sciences Education, Queen Mary University of London, London, UK; 14 2Centre for Health Informatics, Division of Informatics, Imaging and Data Science, Faculty of Biology, Medicine and Health, University of Manchester, Manchester, UK; 15 NIHR Academy, National Institute for Health & Care Research, Leeds, UK; 16 Cardiology Department, Royal Stoke University Hospitals, University Hospitals of North Midlands NHS Trust, Stoke-on-Trent, UK

**Keywords:** E-cigarettes, Vape, Vaping, Electronic nicotine delivery systems, Myocardial infarction, Coronary artery disease, ENDs, Cardiovascular disease, Stroke, Meta-analysis, MACE

## Abstract

**Background:**

Electronic Nicotine Delivery Systems (ENDS), including e-cigarettes, are increasingly used worldwide, yet their association with major adverse cardiovascular outcomes remains inconclusive.

**Methods:**

We conducted a systematic review and meta-analysis (PROSPERO: CRD420251026677) in accordance with PRISMA guidelines. PubMed, MEDLINE, Embase, and Scopus were searched up to May 10, 2025. Eligible studies included adolescents (13–18 years) and adults (≥ 19 years) using ENDS, reporting outcomes on any of the following: non-fatal myocardial infarction (MI), stroke, coronary artery disease (CAD), or cardiovascular mortality. The pooled odds ratios (ORs) were estimated using random-effects models and were considered the primary effect measures for assessing cardiovascular risk across exposure groups. Pooled outcome proportions were reported as secondary measures, reflecting the prevalence of outcomes within the ENDS-exposed population.

**Results:**

Twenty-six studies, comprising over 900,000 ENDS users, were analyzed. The meta-analyses showed significantly higher pooled odds of coronary heart disease (OR = 1.19, *p* < 0.0001), major adverse cardiovascular events (MACE) (OR = 1.57, *p* < 0.0001), and stroke (OR = 1.62, *p* < 0.01). The greatest risk was observed among dual users (OR = 2.21, *p* < 0.0001.), indicating additive cardiovascular harm associated with combined use. All outcomes demonstrated substantial heterogeneity between-study variability.

**Conclusion:**

ENDS use is associated with potential cardiovascular harm, particularly regarding stroke and non-fatal MI; however, the overall findings are limited by substantial heterogeneity. Associations with CAD and mortality remain inconclusive due to data limitations. High-quality longitudinal studies are needed to clarify the long-term cardiovascular risks of ENDS, which could ultimately guide public health policies and initiatives.

**Supplementary Information:**

The online version contains supplementary material available at 10.1186/s12889-026-26302-x.

## Introduction

Cardiovascular diseases (CVDs) remain the leading cause of death worldwide, accounting for nearly one-third of global mortality [1]. Tobacco smoking is a major modifiable risk factor for CVDs. Although traditional cigarette use has declined over recent years [2], this decline has coincided with the introduction of electronic nicotine delivery systems (ENDS) in 2007 and a rapid surge in their use by 2010 [3]. ENDS, including e-cigarettes, e-pipes, and e-hookahs, are now widely used, with global prevalence estimated at around 10% and lifetime prevalence exceeding 20% in some surveys [4]. In the United States, ever-use among adults increased from 12.6% in 2014 to 15.6% in 2016, with especially high uptake among adolescents and young adults [5]. While ENDS expose users to fewer combustion-derived toxins than traditional cigarettes, ENDS still deliver substantial nicotine doses and other potentially harmful constituents [6]. Nicotine increases heart rate, blood pressure, and platelet activation, while aerosolized solvents and flavorings may contribute to oxidative stress, vascular inflammation, and endothelial dysfunction processes implicated in atherothrombosis and adverse cardiovascular outcomes [7–10].

Epidemiologic data increasingly suggest that ENDS use may not be benign. Some studies have reported elevated risks of myocardial infarction among ENDS users compared with non-users, though lower than those observed among conventional smokers [11]. Importantly, such relative risk reduction does not equate to safety, and growing evidence challenges the perception of ENDS as harmless substitutes [12]. Despite this, most research to date has focused on isolated cardiovascular outcomes such as myocardial infarction or stroke, often in cross-sectional or short-term designs. This fragmented evidence base limits insight into the broader impact of ENDS on all major adverse cardiovascular events (MACE). In recent years, a growing proportion of individuals have adopted dual use—concurrently using both electronic nicotine delivery systems (ENDS) and traditional combustible cigarettes, which complicates the evaluation of cardiovascular risks associated with each behavior. Clarifying these associations is essential for policymakers and health systems to guide regulation, preventive strategies, and public health messaging regarding ENDS use.

To address these gaps, we conducted a systematic review and meta-analysis to evaluate the association between ENDS use and major adverse cardiovascular events (MACE). Specifically, we assessed outcomes including non-fatal MI, stroke, coronary artery disease (CAD), and cardiovascular mortality in ENDS users, traditional smokers, non-users, and dual users.

## Methodology

### Study design and registration

This systematic review and meta-analysis were conducted in accordance with the PRISMA (Preferred Reporting Items for Systematic Reviews and Meta-Analyses) guidelines [13]. The study protocol was registered in the International Prospective Register of Systematic Reviews (PROSPERO) under registration number CRD420251026677.

### Information sources and selection process

We performed a comprehensive search of the Embase, Web of Science, PubMed, and Scopus databases, including observational studies (e.g., case-control, cross-sectional, and cohort) published up to May 10, 2025, to evaluate the association between E-cigarettes and major adverse cardiovascular events (MACE). The search strategy was developed using the following search terms: “E-cigarettes”, “Myocardial Infarction”, “Coronary artery disease”, “ENDS”, “cardiovascular disease”, “stroke”, “MACE, “Mortality”. The full search strategy is provided in Supplementary Material File 2, Table S1.

**Fig. 1:**
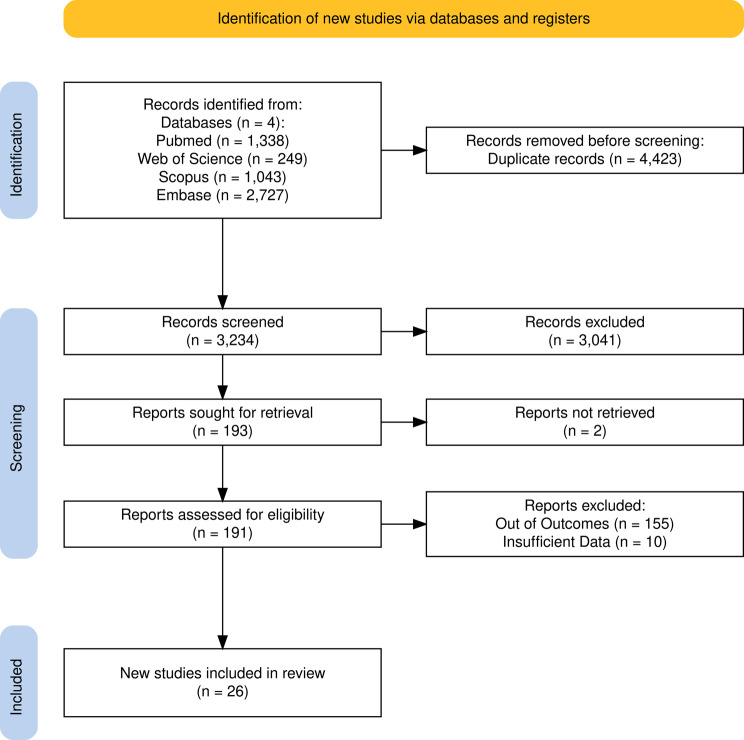
PRISMA flow chart of the included studies

The screening process was split into two stages, conducted by three authors [*A.MA*.E., A.R.A.A., A.A.Z.H.] who independently screened titles and abstracts according to predefined eligibility criteria, with conflicts resolved by a third reviewer [S.N.A.]. Screening was performed using the Rayyan platform [14]. Eligible full-text articles were then reviewed in detail. The study selection process is summarized in the PRISMA flow diagram (Fig. 1).

In addition, we manually screened the reference lists of included studies to identify any additional relevant publications.

### Eligibility criteria

Following the PICOS framework, Population: Adolescent (13–18 years) and adults (≥ 19 years) who use electronic cigarettes, Exposure: Use of electronic cigarettes, Comparator: Non-smokers, Traditional cigarette smokers, Dual users (ENDS + traditional cigarettes), Outcomes of Interest: Major Adverse Cardiovascular Events.

We included studies that meet our included criteria that consisted of: [1] adolescents (13–18 years) and adults (≥ 19 years) using E-cigarettes; [2] Observational studies (case-control, case-series, cross-sectional, and cohort); [3] Studies included having to report at least one of major adverse cardiovascular events that included myocardial infarction (MI), stroke, coronary artery disease (CAD), or cardiovascular mortality; [4] There were no language restrictions (studies in non-English languages will be translated if required); [5] no publication date or geographical location restrictions to ensure comprehensive coverage.

We excluded studies that: [1] Animal studies or in vitro studies; [2] studies not report major adverse cardiovascular events; [3] were Case reports, editorials, letters, or conference abstracts; [4] studies that focused solely on traditional cigarette smoking without ENDS data; [5] studies that related to effects of vaping on cardiovascular physiology rather than outcomes; [7] non-English studies without a translated version available.

### Data extraction and synthesis

Data were extracted independently by two authors [A.R.A.A., A.A.Z.H.] into a standardized online sheet created by the lead author [S.N.A]. Discrepancies were resolved by a third reviewer [S.N.A]. Extraction was structured into three domains:

- Study characteristics: Author, Year, Study design, Country, Sample Size.
- Demographics and baseline characteristics: Age (mean, SD), Gender, Comorbidities, Smoking Status, History of CVD.
- Outcome measures: Non-fatal MI, Stroke, CAD, CV mortality. To harmonize the diverse definitions of ENDS exposure, we standardized users into three primary categories: current (daily/some-day), former, and dual users. In cases where exposure was defined broadly, we utilized the study’s primary definition to maximize data inclusion while acknowledging this variability in our subgroup analyses. A detailed explanation and definitions of these outcomes were reported in Supplementary Material File 2, Table S2.

### Risk of bias and quality assessment

One reviewer [M.A.S] evaluated the risk of bias for each included study and evaluated their quality using the Newcastle Ottawa scale (NOS) [15]. Any discrepancies were reviewed and thoroughly discussed to reach a mutual agreement.

### Effect measures and statistical analysis

For comparative analyses, pooled odds ratios (ORs) with corresponding 95% confidence intervals (CIs) were estimated using random-effects models and were considered the primary effect measures for assessing cardiovascular risk across exposure groups. Pooled outcome proportions with 95% CIs were calculated using the DerSimonian–Laird random-effects model in R (version 4.5.1) with the *meta* package [16] and were reported as secondary measures, reflecting the prevalence of outcomes within the ENDS-exposed population.

Statistical heterogeneity was assessed using the Chi-square test (*p* ≤ 0.10 considered significant) and quantified using the I² statistic (I² = (Q − df)/Q × 100%), with values > 50% indicating substantial heterogeneity. Sensitivity analyses were conducted by sequentially excluding individual studies to assess robustness and the influence of individual studies on pooled estimates. Subgroup meta-analyses compared cardiovascular risk across traditional cigarette users, e-cigarette users, and dual users using pooled ORs.

### Ethical approval

Ethical approval wasn’t required because this study is a systematic review and meta-analysis that utilized previously published data without using patients’ individual data.

## Results

### Selection process

A total of 7,657 records were retrieved from the databases: Scopus (1,043), Web of Science (2,549), PubMed (1,338), and Embase (2,727). After removing 4423 duplicates, 3234 underwent title and abstract screening. Of these, 161 studies were selected for full-text review. Ultimately, 26 studies met the inclusion criteria and were included in our meta-analysis. The PRISMA flowchart outlining the selection process is shown in Fig. 1.

### Study characteristics

Included studies comprised 26 observational studies (primarily cohort and cross-sectional designs), conducted across North America, Europe, and Asia between 2014 and 2025. Most studies originated from the United States (*N* = 24). Sample sizes ranged from *N* = 37 to *N* = 5,159,538, with a cumulative population exceeding 900,000 ENDS users. The mean participant age was generally in the 30 s to 40 s, though some studies included adolescents. Male participants predominated across most cohorts, consistent with reported ENDS use demographics. Dual use of combustible cigarettes was variably reported, while definitions of “ENDS user” differed considerably across studies (current, former, dual). A summary of the included studies is presented in Table 1.

**Table 1:** Summary of all included studies

Author/Year	Study Design	Country	Sample Size	Age (Mean ± SD)	Gender, M/F	Hx of CVD	Smoking Status	Comparator Group	Outcomes
Farfán Bajaña et al., 2024 [17]	Cross-sectional (BRFSS 2020)	USA	198,530	Categories only	50/50	Self-reported MI/CHD	Daily, Some days, Former, Never	Never EC	Former OR 1.23; Some-day OR 1.52; Daily NS
Kang et al., 2025 [18]	Retrospective cohort (K-NHIS, PCI)	South Korea	17,973	60.4	97.1%/3.9%	CAD post-PCI	Continued, Switched, Quitters	Continued smokers	Switchers HR 0.82; Quitters HR 0.87; Exclusive HR 0.71
Ruedisueli et al., 2024 [19]	Randomized crossover	USA	37	40.5 ± 9.1	36/1	Excluded CVD; on ART	Daily smokers	Straw, TC	HR ↑ both EC & TC; LF/HF ↑ less with EC; Nicotine lower
Bricknell et al., 2021 [20]	Cross-sectional (BRFSS 2016)	USA	465,594	Age in categories	NA	CAD, DM, CKD	ENDS (EDU, SDU, FU, NU)	Never EC	EDU OR 1.62; SDU OR 1.28; FU NS
Alzahrani, 2023 [21]	Cross-sectional (NHIS 2014–2021)	USA	148,837	Median 28 EC, 50 never	NA	NA	Never-smokers only	Never EC	OR 2.62 for MI
Falk et al., 2022 [22]	Cross-sectional (NHIS 2014–18)	USA	84,553	> 85% EC < 35 yrs	NA	HTN, Stroke, CAD, DM, MI	6 groups (EC, Comb, Former, Dual, Never)	Never	EC: HTN OR 1.24; Stroke NS; MI NS. Dual: MI OR 3.84
Vindhyal et al., 2020 (NHIS) [23]	Cross-sectional (2016–17)	USA	96,467	Not given	NA	HTN, Stroke, CAD, MI	EC, Comb, Dual, Non	Non	EC: MI OR 1.79, Stroke OR 2.36, CAD OR 1.70, HTN OR 1.72
Parekh et al., 2019 [24]	Cross-sectional (BRFSS 2016–17)	USA	161,529 (18–44 yrs)	68.9% EC 18–24	NA	Stroke	Sole EC, Sole CC, EC + former CC, Dual, Non	Non	Sole EC NS; Sole CC OR 1.59; EC + former CC OR 2.54; Dual OR 2.91
Vindhyal et al., 2020 (Cureus) [23]	Cross-sectional (NHIS 2014–18)	USA	16,855	EC mean 26.7	63%/37%	MI, Stroke, CHD	EC, Comb, Dual, Former Comb ± EC, None	Non	EC: MI OR 4.09 (↑); Stroke NS; CHD NS. Dual: MI OR 5.44; Stroke OR 2.32; CHD OR 2.27
Critcher & Siegel, 2021 [25]	Cross-sectional (NHIS 2014–19)	USA	175,546	Mean 50.6 ± 18.5	46%/54%	MI, comorbidities	EC (ED, SD, Former, Never); Combustible	Never EC	MI ↑ only in current smokers; Daily EC use in never smokers: OR 1.55 (95% CI: 1.11–2.15) under the independent-effects model (artifact). No reliable asso. In never smokers. Annual decline in EC–MI link: AOR 0.81 (95% CI: 0.67–0.98).
Hirschtick et al., 2023 [26]	Longitudinal (PATH, 2013–19)	USA	11,031 (MI); 11,076 (Stroke)	Mean ~ 57.6 (SD 11.7–11.8)	45%/55%	Excluded baseline MI/stroke; adjusted for HTN, DM, pack-years	Non-current, Cigarette, ENDS, Dual	Non-current	Cigarettes ↑ MI (aHR 1.99) & stroke (aHR 2.26); ENDS & dual NS
Naqvi & Searles, 2025 [27]	Cross-sectional (NHANES 2015–18)	USA	11,189	61.9% EC 20–40 yrs	55.5%/44.5%	Self-reported MI, angina, CHF, stroke	Ever EC vs. non; CC included	Non-EC	Angina AOR 3.32; MI AOR 2.60; Stroke NS; CHF NS; sex-specific sig.
Berlowitz et al., 2022 [28]	Longitudinal (PATH 2013–19)	USA	24,027	50% <35 yrs	49%/51%	Excluded baseline CVD	Nonuse, EC only, CC only, Dual	Nonuse	EC HR 1.00 (NS); CC HR 1.53; Dual HR 1.54 (↑); For MI/HF/Stroke: EC HR 1.35 (NS); CC HR 2.20 (↑); Dual HR 2.08 (↑)
Liu, Yuan & Ji, 2022 [29]	Cross-sectional (BRFSS 2020)	USA	253,561	< 35: 27.3%; 35–55: 31.3%; ≥55: 41.4%	49.3%/50.7%	Self-reported MI, CHD, stroke	Never, Former, Current EC; Combustible also categorized	Never EC	Former EC OR 1.108 ↑; Current EC OR 1.170 (NS); Combustible ↑ risk; Insufficient sleep OR 1.592; Excessive OR 1.523; Joint effect (EC + poor sleep) OR 2.596, strongest in middle-aged
Osei et al., 2019 [30]	Cross-sectional (BRFSS 2016–17)	USA	449,092	EC median 30–34; Never EC 45–49	58.8%/41.2%	MI, CHD, Stroke	EC status (never, occasional, daily) stratified by smoker status	Never EC	Dual use of e-cigarettes + combustible cigarettes was associated with 36% higher odds of cardiovascular disease compared with smoking alone (OR 1.36; 95% CI 1.18–1.56); daily dual users had the highest risk (OR 1.59; 95% CI 1.20–2.08). No significant association observed among never-smokers.
Shi et al., 2023 [31]	Cross-sectional (NHANES 2017–18)	USA	4022	Median 55 (IQR 43–65)	48.3%/51.2%	Stroke	Non, Comb, EC, Dual	Nonsmokers	Sole EC OR 2.07; Sole Comb OR 2.36; Dual OR 2.34; Random forest AUC 0.74
Plurphanswat et al., 2024 [32]	Cross-sectional replication (NHIS 2014–21)	USA	132,603	Median 24 EC vs. 50 never	NA	MI, CHD, Stroke	Current EC, Never, Former triers	Never EC	MI crude OR 0.42 → adj OR 2.48; effect driven by age; CHD OR 1.12 (NS); Stroke OR 1.13 (NS)
Mahoney et al., 2022 [33]	Longitudinal (PATH W1–W5)	USA	7820 (10,548 obs)	≥ 40 yrs; mean 52–58	NA	Excluded baseline CVD	Combustible, Switch ENDS, Dual, Quit, Never	Never users	Combustible AOR 1.44 (NS); Switch ENDS 0 cases; Dual AOR 1.85 (NS); Quit AOR 1.18 (NS)
Farsalinos et al., 2019 [34]	Cross-sectional (NHIS 2016–17)	USA	~ 59,770 (2016 & 2017 combined)	Not reported	NA	Adjusted for CVD risk factors	Some days & daily e-cigarette use	Never e-cigarette users	MI: 2017 some-days use OR 2.11; pooled daily use non-significant. CHD: 2016 daily use OR 1.89; pooled non-significant.
Patel et al., 2022 [35]	Cross-sectional (NHANES 2015–18)	USA	79,825 smokers (7,756 EC; 23,444 dual; 48,625 traditional)	Stroke onset: EC 48 yrs; traditional 59 yrs	63.6%/36.4%	Stroke	Exclusive EC, Traditional, Dual	Traditional smokers	Stroke prevalence: EC 1.57%, dual 3.72%, traditional 6.75%; EC earlier onset; adj OR EC vs. traditional 1.15; dual vs. traditional 1.14; EC last 30 d aOR 1.60
Elo-Eghosa et al., 2024 [36]	Cross-sectional (BRFSS 2020–22)	USA	480,317	18–54 yrs	49.9%/50.1%	Premature ASCVD (MI, CHD, Stroke)	Nonuse, former/current EC, former/current CC, dual patterns	Nonuse	CC ↑ risk (aOR 1.68); Dual ↑ risk (aOR 2.03); EC alone NS (aOR ~ 0.9); sex-specific stronger in women (cigs/dual) and men (cig + former EC)
Choi et al., 2021 [37]	Nationwide cohort (NHIS)	South Korea	5,159,538	Mean 41–54 yrs	100%/0%	Excluded baseline CVD; MI/stroke outcome	CC-only, CC + NNTP, recent quit ± NNTP, long-term quit ± NNTP, never	CC-only smokers (and matched quitters)	CC + NNTP aHR 0.83; Recent quit + NNTP aHR 0.81; Long-term quit + NNTP aHR 0.77; But vs. quit w/o NNTP: recent quit aHR 1.31, long-term quit aHR 1.70
Pericot-Valverde et al., 2019 [38]	Cross-sectional (hospitalized cardiac pts)	USA	168	Mean 54.2 ± 12.8	63.7%/36.3%	All had an acute cardiac event	Combusted only vs. combusted + non-combusted	Combusted-only smokers	13.7% used non-combusted too; use higher in younger pts and those with low harm perception; CART: risk ranged 0–47% depending on profile
Qeadan et al., 2023 [39]	Longitudinal (PATH W1–W5)	USA	18,893	Median 41.1 yrs (IQR 27.3)	45.7%/54.4%	Baseline 40.2% CVD (HBP, cholesterol)	ENDS only, Drugs, Dual, Neither	Neither	ENDS only NS (aOR 1.02); Drugs only ↑ CVD (aOR 1.24); Dual ENDS + drugs ↑ respiratory (aOR 1.52) & stroke (aOR 2.48); women dual ↑ resp (aOR 1.63) & CVD (aOR 1.25)
Wang et al., 2018 [40]	Cross-sectional (Health eHeart baseline survey)	USA (mostly)	39,747 (573 EC; 1,693 CC; 514 dual)	Median age: EC 41; CC 45; Dual 46	NA	Self-reported CAD, MI, arrhythmia, CHF, stroke, etc.	No use, EC only, CC only, Dual	No use (ref); also CC-only vs. Dual	Dual use: ↑ cigs/day, worse health & breathing, ↑ arrhythmia (17.8% vs. 14.2%); EC-only vs. no use: worse health, ↑ chest pain, palpitations, CAD, arrhythmia, COPD, asthma
Goldberg Scott et al., 2023 [41]	Cross-sectional + longitudinal (KPRB 2015–19)	USA	119,593	22% ≥70 yrs; younger users ↑ odds	40%/60%	Former ENDS use assoc. w/MI, stroke; current ENDS not	Current, Former, Never ENDS	Never ENDS	Current ENDS: ↑ COPD (OR 2.16), non-stroke CVD (OR 1.55), lung cancer (OR 2.64), ER visits (HR 1.17), death (HR 1.84). Former ENDS: ↑ MI, stroke, COPD, asthma; ↑ hospitalization (HR 1.24). No sig. MI/stroke incidence.

*Abbreviations*:*EC*/*ENDS* Electronic cigarettes/Electronic nicotine delivery systems, *CC*/*Combustible* Combustible cigarettes, *BRFSS* Behavioral Risk Factor Surveillance System, *NHIS* National Health Interview Survey, *NHANES* National Health and Nutrition Examination Survey, *PCI* Percutaneous Coronary Intervention, *CAD* Coronary artery disease, *CHD* Coronary heart disease, *MI* Myocardial infarction, *HF* Heart failure, *HTN* Hypertension, *DM* Diabetes mellitus, *CKD* Chronic kidney disease, *ART* Antiretroviral therapy, *CVD* Cardiovascular disease, *NS* Not significant, *OR* Odds ratio, *AOR* Adjusted odds ratio, *HR* Hazard ratio, *aHR* Adjusted hazard ratio, *SD* Standard deviation, *IQR* Interquartile range, *Comb* Combination / dual use (EC + CC), *EDU* Exclusive daily use (ENDS), *SDU* Some-day use (ENDS), *FU* Former use (ENDS), *NU* Never use (ENDS), *NA* Not Applicable

### Outcomes meta-analysis

**Fig. 2:**
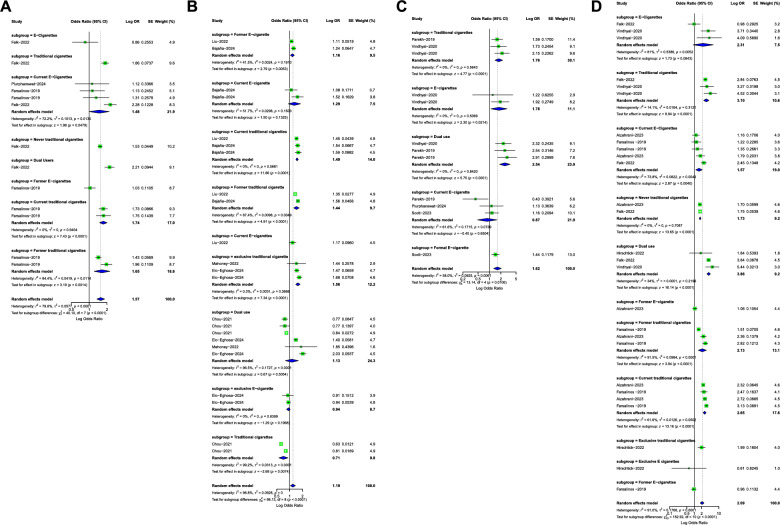
Forest plot showing pooled estimates of the Subgroup of Cardiovascular Clinical Outcomes Risk Among Traditional Cigarettes and E-Cigarettes groups. Each study’s effect size (Odds Ratio) with 95% confidence intervals is displayed, along with overall pooled estimates and heterogeneity statistics (I² = 100%) : (**A**) CAD; (**B**) Major Adverse Cardiovascular Events (MACE); (**C**) Stroke, (**D**) Myocardial Infarction

**Fig. 3:**
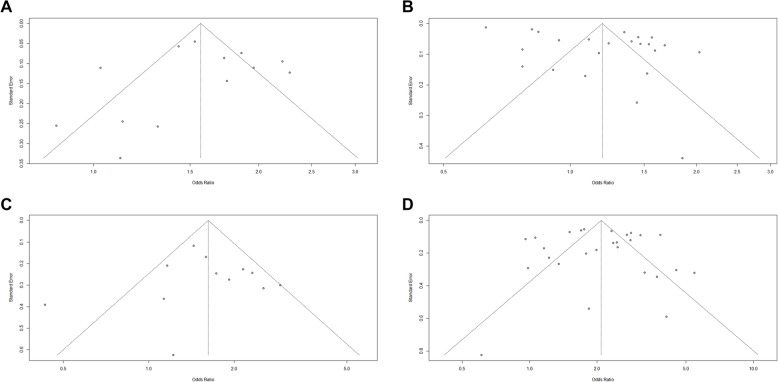
Funnel plots for the assessment of publication bias among studies reporting MACE associated with vaping use: (**A**) CAD; (**B**) Major Adverse Cardiovascular Events (MACE); (**C**) Stroke, (**D**) Myocardial Infarction

Odds Ratio of Cardiovascular Outcomes in identifying risk of CHD among Traditional Cigarettes and E-Cigarettes groups. The pooled OR was 1.19 (*p* < 0.0001), indicating a statistically significant increase in the odds of CHD in all these cigarette classes with significant heterogeneity. The pooled OR of former e-cigarette and current e-cigarette were 1.16 and 1.29, respectively, while the ORs of current traditional cigarettes and former traditional cigarettes were 1.49 and 1.44. The pooled risk was higher in exclusive traditional cigarettes (1.56). The odds ratio was low in exclusive e-cigarette (0.94) and traditional cigarettes (0.71), as shown in Fig. 2A. The funnel plot (Fig. 3A) showed relative symmetry, indicating a low risk of publication bias.

Odds Ratio of Cardiovascular Outcomes in identifying risk of MACE Among Traditional Cigarettes and E-Cigarettes groups. The pooled OR was 1.57 (*p* < 0.0001), indicating a statistically significant increase in the odds of MACE in all these cigarette classes with significant heterogeneity. The pooled OR Current E-Cigarettes (1.48), Former E-Cigarettes (1.03), Traditional Cigarettes (1.86), and Dual Users (2.21). The pooled risk was higher in Dual cigarettes (2.21), as shown in Fig. 2B. The funnel plot ( Fig. 3B) showed significant asymmetry, indicating possible publication bias.

Odds Ratio of Cardiovascular Outcomes in identifying risk of stroke among Traditional Cigarettes and E-Cigarettes groups. The pooled OR was 1.62 (*p* < 0.0061), indicating a statistically significant increase in the odds of stroke in all these cigarette classes with significant heterogeneity. The pooled OR of current e-cigarette(0.87), traditional cigarettes (2.76), and e-cigarettes(1.78). The pooled risk was higher in dual cigarettes (2.54), as shown in Fig. 2C. The funnel plot (Fig. 3C) showed relative symmetry, indicating a low risk of publication bias.

The odds ratios (ORs) of cardiovascular outcomes for identifying the risk of myocardial infarction among traditional cigarette and e-cigarette groups. The overall pooled OR was 2.09, indicating a statistically significant increase in the odds of myocardial infarction across all cigarette classes, with significant heterogeneity (I² = 91.0%, *p* < 0.0001). Subgroup analysis showed pooled ORs of 1.57 for current e-cigarette users, 3.10 for traditional cigarette users, and 2.31 for e-cigarette users overall. The highest pooled risk was observed among dual users of traditional cigarettes and e-cigarettes, with a pooled OR of 3.86, as shown in Fig. 2D. The funnel plot (Fig. 3D) showed high heterogeneity and asymmetry, indicating possible publication bias.

### Prevalence of the outcome

#### Cardiovascular mortality

**Fig. 4:**
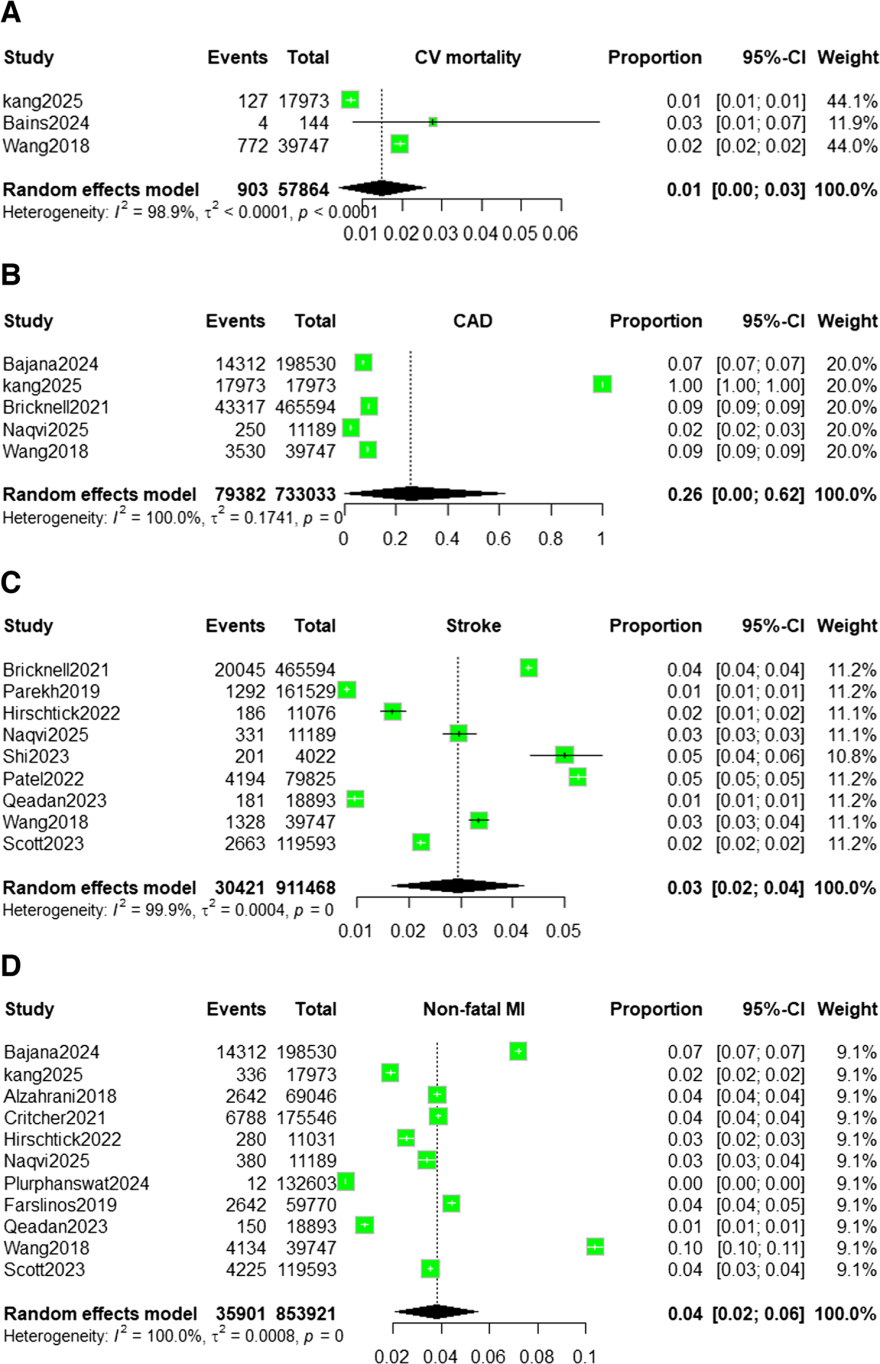
Forest plot showing pooled estimates of MACE among vaping users using a random-effects model. Each study’s effect size (proportion) with 95% confidence intervals is displayed, along with overall pooled estimates and heterogeneity statistics (I² = 100%): (**A**) Cardiovascular Mortality; (**B**) Coronary Artery Disease;(**C**) Stroke; (**D**) Non-Fatal Myocardial Infarction

**Fig. 5:**
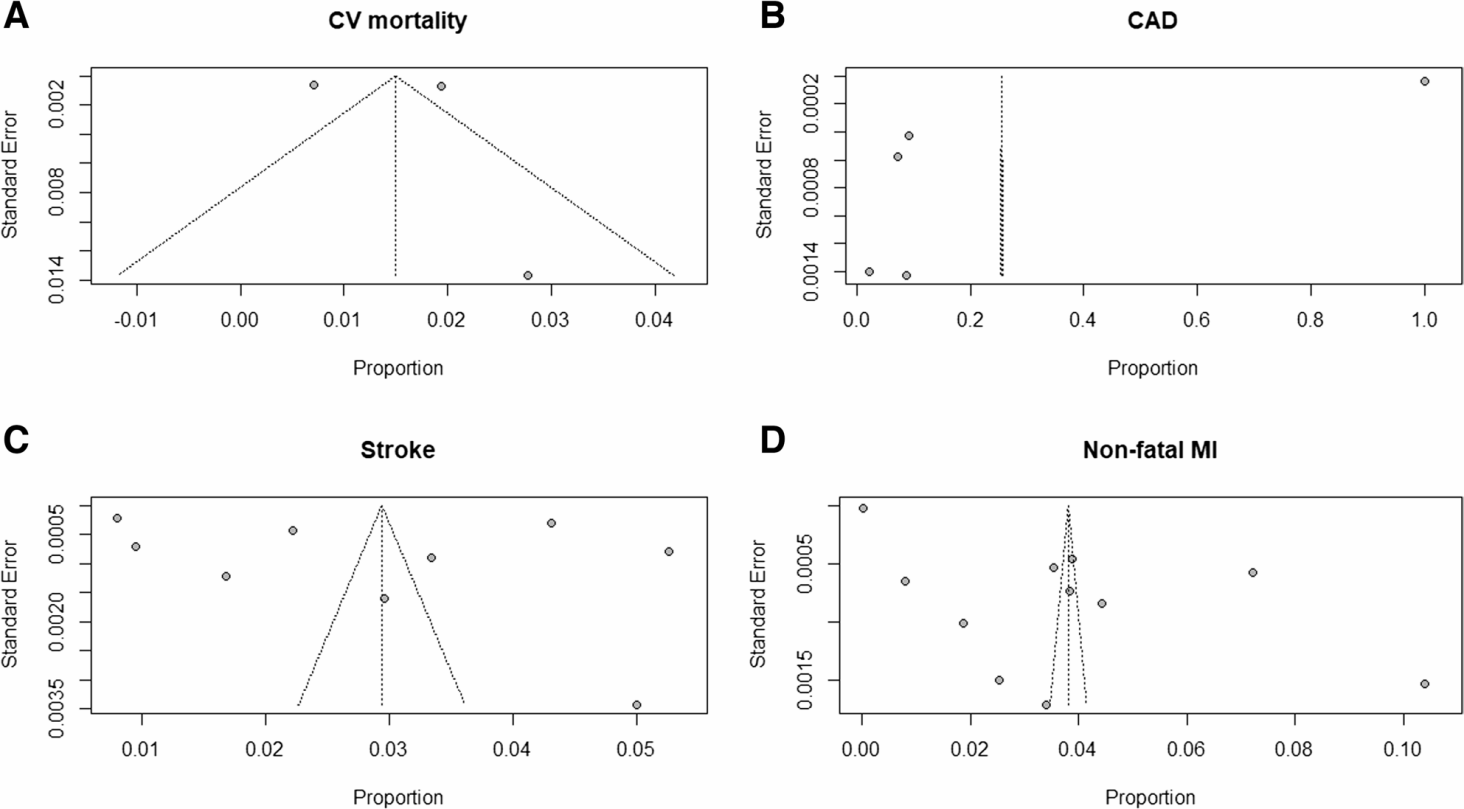
Funnel plots for the assessment of publication bias among studies reporting MACE associated with vaping use: (**A**) Cardiovascular mortality; (**B**) CAD; (**C**) Stroke; (**D**) Non-fatal MI

We analyzed three cohort studies that reported cardiovascular mortality outcomes, yielding 903 deaths among 57,864 ENDS users. Using a random-effects model, the pooled mortality proportion was 0.01 [0.00–0.03.00.03]. Between-study heterogeneity was high (I² = 98.9%, τ² < 0.0001, *p* < 0.0001). Leave-one-out sensitivity analysis demonstrated minimal influence of individual studies. Excluding Kang [18] resulted in a pooled proportion of 0.02 [0.02–0.02] with I² = 0%, as shown in Fig. 4A. The funnel plot (Fig. 5A) showed significant asymmetry, indicating possible publication bias.

#### Coronary Artery Disease (CAD)

We analyzed five cohort studies that contributed a total of 79,382 CAD events among 733,033 ENDS users. Individual study proportions were 0.07 for Bajana [17], 1.00 for Kang [18], 0.09 for Bricknell [20], 0.02 for Naqvi [27], and 0.09 for Wang [40], with each study contributing 20% to the analytic weight. The random-effects model produced a pooled CAD proportion of 0.26 (95% CI: 0.00–0.62.00.62, P-value = 0). Heterogeneity was extreme (I² = 100%, T² = 0.1741, *p* < 0.001), and the wide confidence interval crossing zero indicated no statistical significance at the α = 0.05 level. Leave-one-out sensitivity analysis produced pooled proportions of 0.30 when omitting Bajana [17], 0.07 when omitting Kang [18], 0.30 when omitting Bricknell [20], 0.31 when omitting Naqvi [27], and 0.30 when omitting Wang [40]. Corresponding 95% confidence intervals were wide (0.00–0.95.00.95 in most cases), except when Kang [18] was excluded, yielding a narrower CI of 0.05–0.09, as shown in Fig. 4B. Heterogeneity remained extreme in all iterations (I² ≈ 100%), indicating that no single study fully accounted for the observed variability (Supplemental materials figure). The funnel plot (Fig. 5B) showed significant asymmetry, indicating possible publication bias.

#### Stroke

The results from nine cohort studies analyzing stroke outcomes, comprising 30,421 events among 911,468 ENDS users. Individual study proportions ranged from 0.01 to 0.05, and because of similar sample sizes after transformation, each study contributed approximately 11% of the analytic weight (10.8–11.2%). The random-effects model produced a pooled stroke proportion of 0.03 (95% CI: 0.02–0.04, P-value = 0). As the confidence interval did not include zero, this result was statistically significant at the two-sided α = 0.05 level. Between-study heterogeneity was extreme (I² = 99.9%, T² = 0.0004, *p* < 0.001). Leave-one-out sensitivity analysis demonstrated stability of the estimate, as excluding any single study yielded pooled proportions that remained at 0.03. The corresponding 95% confidence intervals spanned 0.02–0.04 in most scenarios, with a slightly wider interval of 0.01–0.05 when Shi [31], Patel [35], Qeadan [39], or Goldberg Scott [41] were excluded. Heterogeneity metrics (I² ≈ 99% and T between 0.0154 and 0.0219) changed only marginally, confirming that no individual dataset exerted undue influence, as shown in Fig. 4C. The funnel plot (Fig. 5C) showed a moderate degree of asymmetry, indicating possible publication bias.

#### Non-fatal Myocardial Infarction (MI)

Eleven cohort studies (Bajana [17], Kang [18], Alzahrani [21], Critcher [25], Hirschtick [26], Naqvi [27], Plurphanswat [32], Farsalinos [34], Qeadan [39], Wang [40], and Goldberg Scott [41]) reported on non-fatal MI, totaling 35,901 events among 853,921 ENDS users. Individual study proportions ranged from 0% [32] to 10% [40]. Because of variance-based weighting after transformation, each study contributed approximately 9–10% of the total weight (median 9.1%). The random-effects model produced a pooled MI proportion of 0.04 (95% CI: 0.02–0.06). The confidence interval lay entirely above zero, indicating statistical significance at α = 0.05. Between-study heterogeneity was extreme (I² = 100%, T² = 0.0008, *p* < 0.001). Leave-one-out sensitivity analysis did not materially alter the summary estimate, as pooled proportions remained between 0.03 and 0.04 with corresponding 95% confidence intervals of 0.02–0.05 or 0.02–0.06. Heterogeneity remained high across all iterations (I² ≈ 100%, τ = 0.028–0.031), confirming that no single study exerted undue influence on the overall result. For Non-fatal MI, Egger’s Test result: t = 4.41, df = 9, p-value = 0.0017. Bias estimate: 52.7553 (SE = 11.9656) the funnel plot ( Fig. 5D). Therefore, the pooled proportions presented in the current analysis represent prevalence estimates only and should not be interpreted as indicators of risk or causal association, as shown in Fig. 4D.

### Quality assessment

Risk of bias assessment indicated that the cohort studies were overall rated as low to moderate risk, with concerns primarily related to deviations from intended interventions and incomplete outcome data. Observational studies generally scored between 6,7 and 9 out of 9 stars on the Newcastle–Ottawa Scale (NOS), indicating moderate to high risk of bias, largely due to residual confounding, reliance on self-reported ENDS exposure and cardiovascular outcomes, and limited adjustment for prior smoking history. No study was judged to be at low risk across all domains. (Supplementary Material File 1).

## Discussions

This systematic review and meta-analysis evaluated the association between ENDS use and major adverse cardiovascular events (MACE), including cardiovascular mortality, CAD, stroke, and non-fatal myocardial infarction (MI). Meta-analysis showed a significantly increased risk of cardiovascular outcomes among traditional cigarette and e-cigarette users. The pooled OR for coronary heart disease was 1.19 (*p* < 0.0001), with the highest risk among exclusive traditional smokers (OR = 1.56). Major adverse cardiovascular events were also increased (pooled OR = 1.57, *p* < 0.0001), particularly among dual users (OR = 2.21). Stroke risk was elevated (pooled OR = 1.62, *p* = 0.0061), mainly among traditional cigarette users (OR = 2.76), while current e-cigarette use showed no significant association (OR = 0.87).

These findings generally align with growing clinical evidence [42–44]. The pooled proportion for cardiovascular mortality was lower at 1%, while CAD showed no statistically significant association due to extreme heterogeneity and wide confidence intervals. However, it is important to note that most included studies were cross-sectional in nature, limiting the ability to infer causality. These findings should therefore be interpreted as indicative of potential associations rather than definitive causal relationships.

Several biological mechanisms may underlie these associations. Nicotine, the primary component of most e-liquids, is a potent sympathomimetic that acutely increases heart rate, blood pressure, and myocardial contractility, thereby elevating oxygen demand [9, 45]. Chronic nicotine exposure can induce endothelial dysfunction, a precursor to atherosclerosis, and contribute to oxidative stress and systemic inflammation [13, 46, 47]. Beyond nicotine, ENDS aerosols contain particulate matter (PM2.5), aldehydes such as formaldehyde and acrolein, and volatile organic compounds, many of which are established cardiotoxins [48, 49]. Experimental studies demonstrate that these compounds can promote platelet activation, vascular injury, and inflammatory cascades, all implicated in the pathogenesis of MI and stroke [50–53]. Our findings are consistent with prior large-scale cross-sectional analyses and meta-analyses reporting associations between ENDS use and self-reported MI and stroke [11, 28, 54, 55].

The pooled cardiovascular mortality proportion of 1%, derived from three cohort studies, represents a clinically important endpoint. However, the limited number of studies and high heterogeneity (I² = 98.9%) necessitate cautious interpretation. Mortality is a long-term outcome that may not yet be fully captured, given that widespread ENDS use is relatively recent [56, 57]. Current longitudinal studies may underestimate the true long-term risk, as most have short follow-up durations [42, 44, 58]. Thus, while our data suggest a possible mortality signal, definitive conclusions require larger, long-term prospective studies.

Our CAD analysis was particularly affected by heterogeneity. The pooled estimate of 26% (95% CI: 0.00–0.62.00.62) was not statistically significant, largely due to the influence of one study [18] that reported an implausibly high proportion (1.00). This finding likely reflects a methodological or reporting anomaly, such as a study population composed entirely of CAD patients who were also ENDS users. Leave-one-out analysis confirmed this distortion, showing that removal of Kang [18] yielded a statistically significant pooled estimate of 7% (95% CI: 0.05–0.09). Variability in CAD definitions and ascertainment across studies likely further contributed to instability [34, 59]. These findings highlight how single outlier studies can disproportionately influence pooled results in the setting of extreme heterogeneity.

The extreme heterogeneity observed across all analyses (I² > 98%) is a major limitation and underscores the challenges of synthesizing evidence on ENDS. Several factors likely contributed. First, definitions of “ENDS user” varied widely, with some studies combining daily intensive users with occasional or experimental users [40]. Second, study populations were heterogeneous, often including current smokers, former smokers, dual users, and smoking-naïve individuals [46, 60]. Disentangling the cardiovascular risk attributable to ENDS alone, independent of residual risk from past smoking or dual use, remains a major methodological challenge [61, 62]. Third, the ENDS market itself is dynamic and highly variable. Devices range from early-generation “cig-a-likes” to modern pod-based systems such as JUUL, each delivering nicotine and chemical byproducts differently [63–66]. E-liquid formulations vary in nicotine concentration and flavoring composition, with certain flavorings shown to have unique cytotoxic and pro-inflammatory properties [66, 67]. Most included studies did not stratify analyses by device type, nicotine strength, or flavoring, limiting comparability. Finally, differences in study design, outcome ascertainment (self-reported vs. medical records), follow-up duration, and adjustment for confounders such as diet, physical activity, and socioeconomic status further complicated synthesis [66, 67].

While the absolute proportions of MI (4%) and stroke (3%) among ENDS users appear relatively small, their public health implications are profound. Given the global expansion of the e-cigarette market, these figures suggest that millions of users may be at an elevated risk for major adverse cardiovascular events (MACE), necessitating stricter regulatory oversight and continued clinical surveillance.

A critical consideration in interpreting our findings is the potential for reverse causality, particularly in cross-sectional surveys like BRFSS and NHIS. It is plausible that individuals who have already experienced a cardiovascular event (e.g., MI or Stroke) or have been diagnosed with CAD are more likely to switch from traditional cigarettes to ENDS in an effort to reduce harm. This ‘healthy smoker’ switch could lead to an overestimation of the association between ENDS use and MACE. While our subgroup analysis attempted to differentiate between exclusive users and dual users, the lack of longitudinal data in most studies means that the temporal sequence of ENDS initiation and cardiovascular onset remains unclear. Future prospective cohort studies are essential to disentangle these effects.

Taken together, these findings suggest that ENDS use is associated with an increased risk of non-fatal MI and stroke, while associations with CAD and cardiovascular mortality remain inconclusive. The consistently high heterogeneity across studies highlights the need for well-designed longitudinal research that carefully defines exposure, distinguishes between exclusive ENDS users and dual users, and accounts for device variability and long-term follow-up.

Our study has several limitations. First, the majority of the included studies utilized a cross-sectional design, which captures exposure and outcome simultaneously, thus preventing the establishment of a temporal relationship or causality. Second, the reliance on self-reported cardiovascular events (MI and stroke) introduces a high risk of misclassification bias. While such surveys are vital for large-scale population health monitoring, they may suffer from recall bias. Third, the extreme heterogeneity (I^2^ > 98%) observed across outcomes suggests that the pooled estimates should be viewed with caution, reflecting diverse study populations and varying definitions of ENDS exposure.

The assessment of ENDS exposure relied heavily on self-reported survey data, which is susceptible to misclassification bias. Variations may introduce heterogeneity and potentially bias the pooled estimates toward the null if light users are grouped with heavy users. This limitation underscores the need for more standardized exposure metrics in future tobacco research.

## Conclusion

This systematic review and meta-analysis demonstrate that ENDS use is associated with measurable cardiovascular risk, most notably higher proportions of non-fatal myocardial infarction and stroke. While signals for coronary artery disease and cardiovascular mortality were identified, the current evidence is too inconsistent to allow firm conclusions.

The overarching limitation of existing research lies in methodological heterogeneity ranging from inconsistent definitions of ENDS use to reliance on cross-sectional data and inadequate adjustment for dual use and prior smoking. These limitations reflect the early stage of ENDS research and highlight the need for more rigorous prospective studies.

From a clinical and public health perspective, our findings reinforce that ENDS should not be assumed to be a safe alternative to combustible cigarettes. The observed associations with major cardiovascular events underscore the importance of regulatory oversight, patient counseling, and continued surveillance of emerging products. Future research should prioritize standardized exposure definitions, device- and formulation-specific analyses, and long-term follow-up to better characterize the cardiovascular trajectory of ENDS users.

## Supplementary Information

Supplementary Material 1.

## Data Availability

The datasets used and/or analyzed during the current study are available from the corresponding author on reasonable request.

## Abbreviations

AHR: Adjusted Hazard Ratio
AOR: Adjusted Odds Ratio
ART: Antiretroviral Therapy
ASCVD: Atherosclerotic Cardiovascular Disease
BMI: Body Mass Index
BRFSS: Behavioral Risk Factor Surveillance System
CAD: Coronary Artery Disease
CC: Combustible Cigarette
CHD: Coronary Heart Disease
CI: Confidence Interval
CKD: Chronic Kidney Disease
COPD: Chronic Obstructive Pulmonary Disease
CVD: Cardiovascular Disease
DM: Diabetes Mellitus
EC: E-Cigarette
ENDS: Electronic Nicotine Delivery Systems
ER: Emergency Room
HF: Heart Failure
HR: Hazard Ratio
HTN: Hypertension
I²: I-Squared Statistic (Measure of Heterogeneity)
JUUL: Pod-Based E-Cigarette Brand (JUUL Labs Inc.)
K-NHIS: Korean National Health Insurance Service
MACE: Major Adverse Cardiovascular Events
MI: Myocardial Infarction
NA: Not Applicable
NHANES: National Health and Nutrition Examination Survey
NHIS: National Health Interview Survey
NIHR: National Institute for Health and Care Research
NOS: Newcastle–Ottawa Scale
NS: Not Significant
OR: Odds Ratio
PATH: Population Assessment of Tobacco and Health Study
PCI: Percutaneous Coronary Intervention
PM2.5: Particulate Matter ≤2.5 Micrometers
PRISMA: Preferred Reporting Items for Systematic Reviews and Meta-Analyses
PROSPERO: International Prospective Register of Systematic Reviews
SD: Standard Deviation
TC: Traditional Cigarette
τ² (Tau-Squared): Between-Study Variance (in Meta-Analysis)
WHO: World Health Organization
