# Supplementary material for: Electronic cigarettes and cardiovascular outcomes: a systematic review and meta-analysis of Major Adverse Cardiovascular Events (MACE)
Source: BMC Public Health. 2026 Feb 23;26:704. doi: 10.1186/s12889-026-26302-x (PMC12927215; doi:10.1186/s12889-026-26302-x)
Supplement: Supplementary file 1 — Supplementary Material 1. [file 12889_2026_26302_MOESM1_ESM.docx]

**Supplementary Material:**

**File 1: Risk of bias assessments for included studies.**

| **Newcastle-Ottawa Scale (NOS) for Cohort Studies _Criteria** |
| --- |
| **Selection**  1. Representativeness of the exposed cohort.  2. Selection of the non-exposed cohort.  3. Ascertainment of exposure.  4. Demonstration that the outcome of interest was not present at the start of the study.  **Comparability of cohorts on the basis of the design or analysis**  A study controls for the most important factor.  B.study controls for any additional factor.  **Outcome**  1. Assessment of outcome.  2. Was the follow-up long enough for outcomes to occur?  3. Adequacy of follow-up of cohorts.  **Response Options:** Yes, No, Unclear, Not Applicable (NA).  **Quality Rating:** Low Quality 0–3; Moderate Quality 4–6; High Quality 7–9 |

**
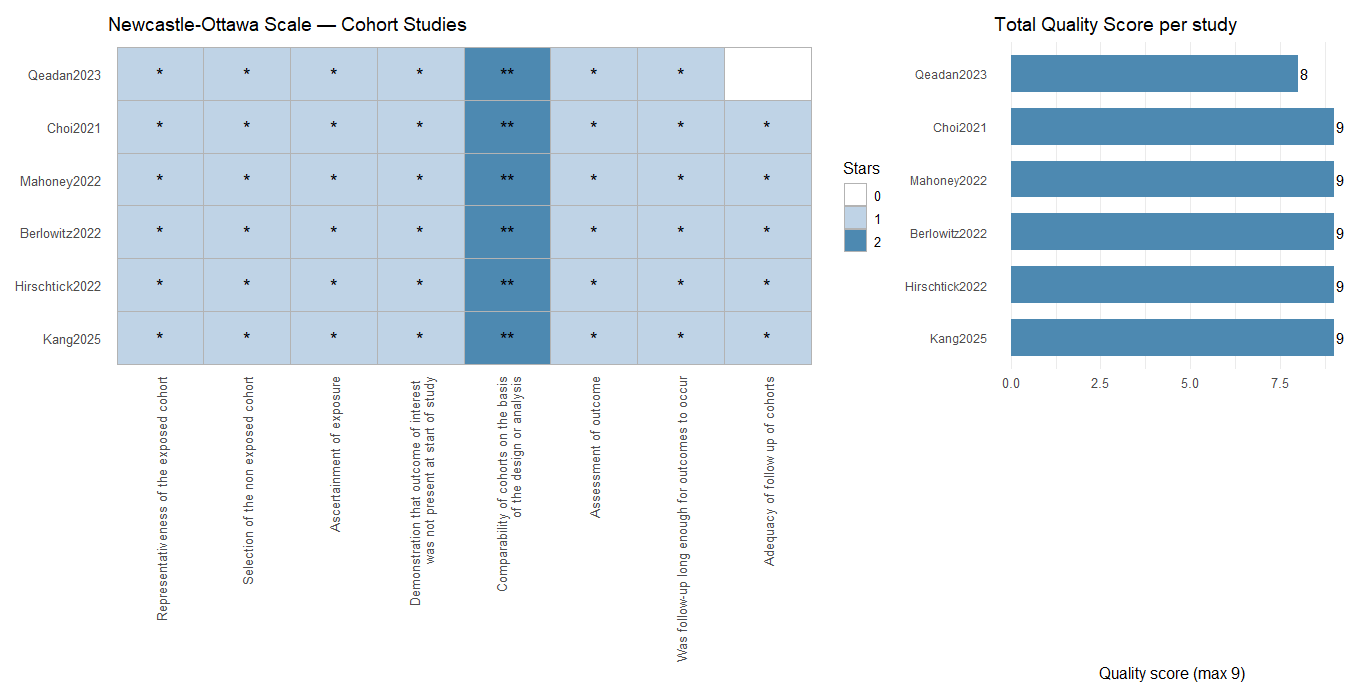
**

**
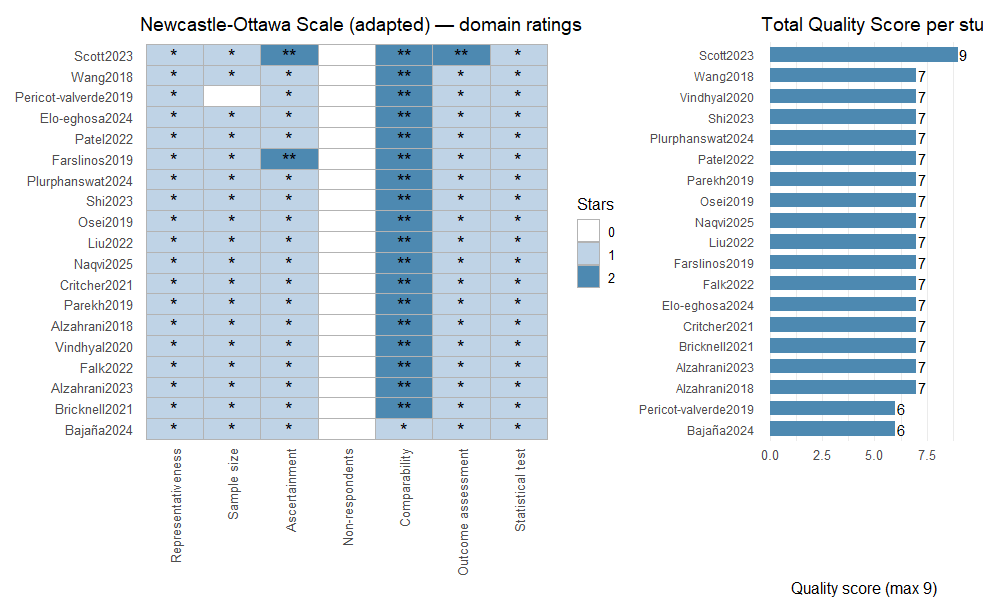
**

**Supplementary Material File 2**

**Table S1: Search term strategy.**

| Database | String | Results |
| --- | --- | --- |
| PubMed | (("Electronic Nicotine Delivery Systems" OR "Electronic Cigarettes" OR "E-cigarette*" OR "Electronic cigarette*" OR "Vaping" OR "Vape" OR "E-smoking" OR "Electronic nicotine delivery system*" OR "Pod-based e-cigarette*" OR "JUUL" OR "Dual use") AND ("Cardiovascular Diseases" OR "Myocardial Infarction" OR "MI"  OR "MACE" OR "Major Adverse Cardiovascular Event*" OR "Coronary Artery Disease" OR "CAD" OR "Acute Coronary Syndrome*" OR "Heart Attack*" OR "Cardiac Arrest" OR "Atherosclerosis" OR "Heart Disease*" OR "Stroke" OR "Arrhythmia*" OR "Cardiovascular Health" OR "Cardiovascular Outcome*" OR "Cardiovascular Mortality" OR "Heart Failure")) | 1338 |
| Web of Science | (("Electronic Nicotine Delivery Systems" OR "Electronic Cigarettes" OR "E-cigarette*" OR "Electronic cigarette*" OR "Vaping" OR "Vape" OR "E-smoking" OR "Electronic nicotine delivery system*" OR "Pod-based e-cigarette*" OR "JUUL" OR "Dual use") AND ("Cardiovascular Diseases" OR "Myocardial Infarction" OR "MI"  OR "MACE" OR "Major Adverse Cardiovascular Event*" OR "Coronary Artery Disease" OR "CAD" OR "Acute Coronary Syndrome*" OR "Heart Attack*" OR "Cardiac Arrest" OR "Atherosclerosis" OR "Heart Disease*" OR "Stroke" OR "Arrhythmia*" OR "Cardiovascular Health" OR "Cardiovascular Outcome*" OR "Cardiovascular Mortality" OR "Heart Failure")) | 2549 |
| Scopus | (("Electronic Nicotine Delivery Systems" OR "Electronic Cigarettes" OR "E-cigarette*" OR "Electronic cigarette*" OR "Vaping" OR "Vape" OR "E-smoking" OR "Electronic nicotine delivery system*" OR "Pod-based e-cigarette*" OR "JUUL" OR "Dual use") AND ("Cardiovascular Diseases" OR "Myocardial Infarction" OR "MI"  OR "MACE" OR "Major Adverse Cardiovascular Event*" OR "Coronary Artery Disease" OR "CAD" OR "Acute Coronary Syndrome*" OR "Heart Attack*" OR "Cardiac Arrest" OR "Atherosclerosis" OR "Heart Disease*" OR "Stroke" OR "Arrhythmia*" OR "Cardiovascular Health" OR "Cardiovascular Outcome*" OR "Cardiovascular Mortality" OR "Heart Failure")) | 1043 |
| Embase | (("Electronic Nicotine Delivery Systems" OR "Electronic Cigarettes" OR "E-cigarette*" OR "Electronic cigarette*" OR "Vaping" OR "Vape" OR "E-smoking" OR "Electronic nicotine delivery system*" OR "Pod-based e-cigarette*" OR "JUUL" OR "Dual use") AND ("Cardiovascular Diseases" OR "Myocardial Infarction" OR "MI"  OR "MACE" OR "Major Adverse Cardiovascular Event*" OR "Coronary Artery Disease" OR "CAD" OR "Acute Coronary Syndrome*" OR "Heart Attack*" OR "Cardiac Arrest" OR "Atherosclerosis" OR "Heart Disease*" OR "Stroke" OR "Arrhythmia*" OR "Cardiovascular Health" OR "Cardiovascular Outcome*" OR "Cardiovascular Mortality" OR "Heart Failure")) | 2727 |

**Table S2.** Definitions of exposure and outcomes assessed in the included studies.

| **Exposure/Outcomes** | **Definitions** |
| --- | --- |
| **ENDS (Electronic Nicotine Delivery Systems)** | Devices that deliver nicotine in an aerosolized form without combustion, including e-cigarettes, vape pens, and similar electronic vaping products. |
| **Current E-cigarettes** | Participants who currently use e-cigarettes on some days or every day. |
| **Former E-cigarettes** | Participants who had used e-cigarettes in the past but no longer use them. |
| **Never E-cigarettes** | Participants who have never used an e-cigarette. |
| **Current Traditional cigarettes** | Participants who currently smoke combustible cigarettes on some days or every day. |
| **Former Traditional cigarettes** | Participants who smoked in the past but no longer smoke. |
| **Never Traditional cigarettes** | Participants who have never smoked 100 cigarettes in their lifetime. |
| **Dual Cigarettes** | Participants who currently use both e-cigarettes and combustible cigarettes. |
| **MACE** | Major adverse cardiovascular events (composite outcome including MI, stroke, and cardiac death). Calculated as event number, total participants, and effect estimates (OR/HR with 95% CI). |
| **Cardiovascular Mortality** | Death specifically due to cardiac causes. Calculated as event number, total participants, and effect estimates (OR/HR with 95% CI). |
| **Myocardial Infarction (MI)** | Self-reported or adjudicated MI. Calculated as event number, total participants, and effect estimates (OR/HR with 95% CI) |
| **Coronary Artery Disease (CAD)** | Incident or prevalent CAD events. Calculated as event number, total participants, and effect estimates (OR/HR with 95% CI). |
| **Stroke** | Incident or prevalent Stroke events. Calculated as event number, total participants, and effect estimates (OR/HR with 95% CI). |


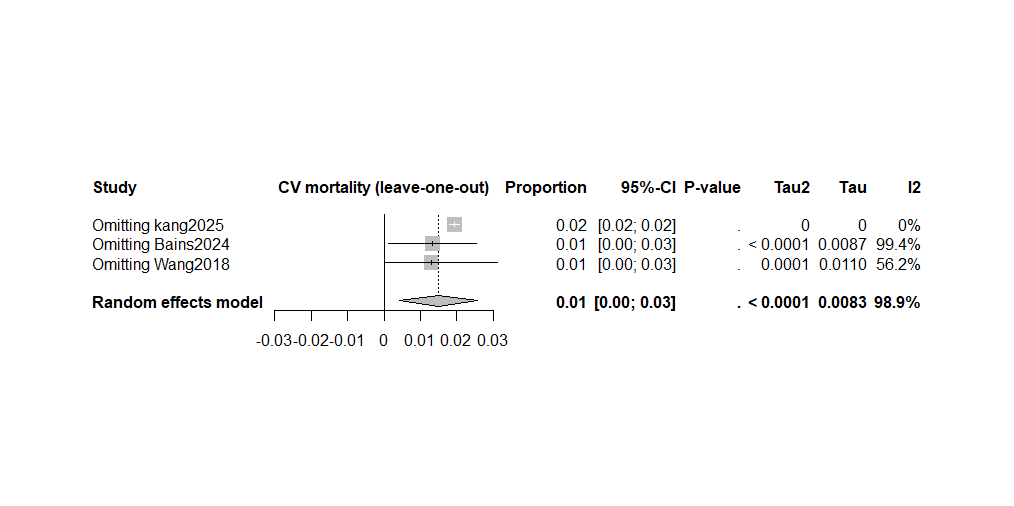
**Figure S1:** Leave one analysis: Sensitivity analysis of pooled cardiovascular mortality estimates after sequential omission of individual studies in the meta-analysis of vaping-related cardiovascular mortality outcomes.


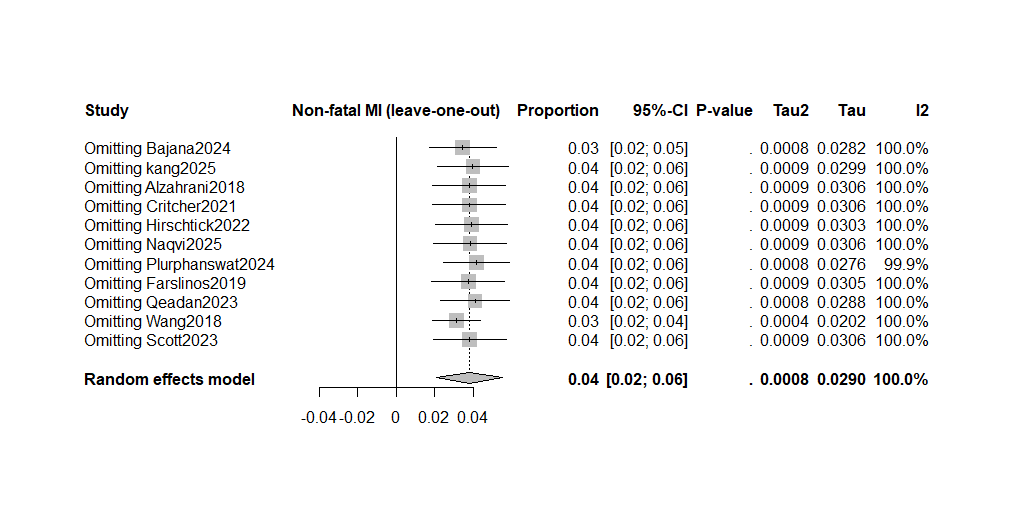
**Figure S2:** Leave one analysis: Sensitivity analysis of pooled Non-fatal MI estimates after sequential omission of individual studies in the meta-analysis of vaping-related Non-fatal MI outcomes.


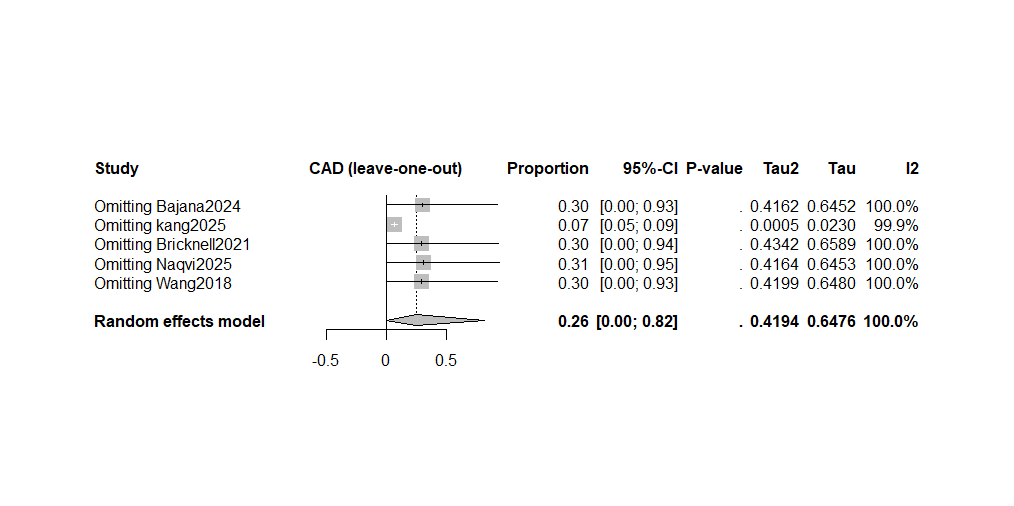
**Figure S3:** Leave one analysis: Sensitivity analysis of pooled CAD estimates after sequential omission of individual studies in the meta-analysis of vaping-related CAD outcomes.


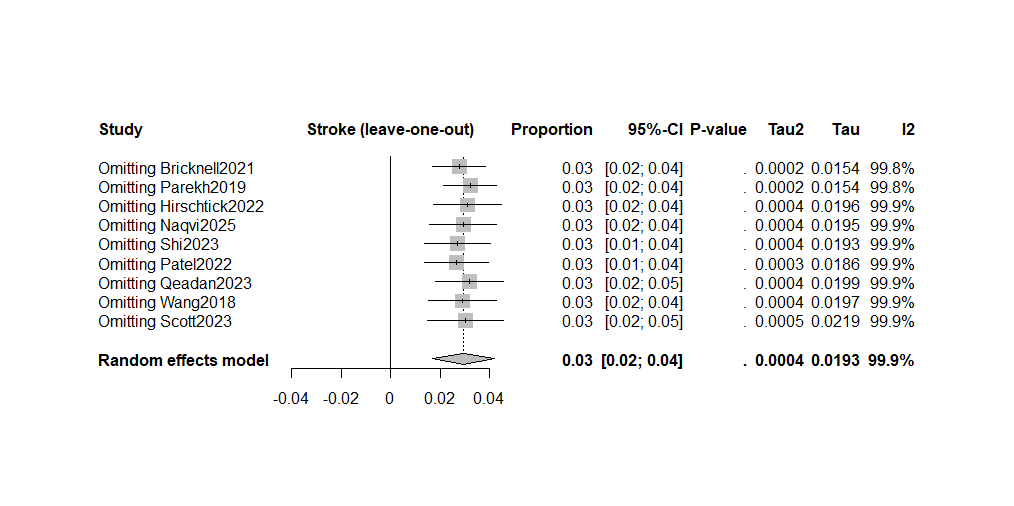
**Figure S4:** Leave one analysis: Sensitivity analysis of pooled Stroke estimates after sequential omission of individual studies in the meta-analysis of vaping-related stroke outcomes.
